# A case of Incontinentia Pigmenti associated with concurrent *IKBKG/NEMO* and *MED13L* mutations

**DOI:** 10.3389/fmed.2026.1819035

**Published:** 2026-06-18

**Authors:** Ezia Spinosa, Jeremie Rosain, Stefania Picascia, Michele Salvia, Alessandra Pescatore, Annalaura Torella, Giulio Piluso, Vincenzo Nigro, Vincenzo Piccolo, Andrea Diociaiuti, Immacolata Di Biase, May El Hachem, Maria B. Lioi, Paul Bastard, Matilde V. Ursini, Francesca Fusco

**Affiliations:** 1 Institute of Genetics and Biophysics “Adriano Buzzati- Traverso” IGB-CNR, Naples, Italy; 2 Laboratory of Human Genetics of Infectious Diseases, Necker Branch, INSERM U1163, Necker Hospital for Sick Children, Paris, France; 3 Imagine Institute, University of Paris Cité, Paris, France; 4 Department of Precision Medicine, The University of Campania “Luigi Vanvitelli”, Naples, Italy; 5 Telethon Institute of Genetics and Medicine (TIGEM), Naples, Italy; 6 Dermatology Unit, University of Campania “Luigi Vanvitelli”, Naples, Italy; 7 Dermatology Unit and Genodermatosis Research Unit, Translational Paediatrics and Clinical Genetics Research Division, Bambino Gesù Children’s Hospital, IRCCS, Rome, Italy; 8 MeriGen Diagnostic & C. SAS, Naples, Italy; 9 Department of Basic and Applied Sciences, University of Basilicata, Potenza, Italy; 10 Pediatric Hematology-Immunology and Rheumatology Unit, Necker Hospital for Sick Children, Assistance Publique-Hôpitaux de Paris (AP-HP), Paris, France

**Keywords:** IKBKG/NEMO, impaired intellectual development and distinctive facial features with or without cardiac defects, Incontinentia Pigmenti, IP mosaicism, *MED13L*

## Abstract

Incontinentia Pigmenti (IP; OMIM#308300) and syndromic intellectual disability (ID) (MRFACD; OMIM#616789) are two genetically dominant rare diseases. Their phenotypes are characterized by distinctive clinical signs: IP is caused by skin and neuroectodermal abnormalities with highly variable expression, while MRFACD is caused by a broad range of neurologic manifestations, including ID, hypotonia, ophthalmological abnormalities, motor delay, abnormalities in cerebral magnetic resonance, and a remarkable speech delay. The two diseases have genetically different causes: IP is an X-linked disorder caused by mutations in the *IKBKG/NEMO* gene, whereas MRFACD is an autosomal dominant disease caused by mutations in the *MED13L* gene. In this study, we describe the unique case of a female patient with a complex phenotype characterized by neuroectodermal abnormalities typical of IP and by syndromic intellectual disability. The multiple genetic approaches revealed the concurrence of postzygotic mosaicism for the genomic deletion (NEMOdelta4_10) in the *IKBKG* gene and of a constitutive deleterious variant in the *MED13L* gene (NM_015335.4: c.1708_1709del). This genetic combination, never previously reported, makes this case particularly interesting from a clinical perspective because it underscores the importance of considering multilocus genomic alterations, including postzygotic mosaicism, as possible contributors to complex clinical presentations.

## Introduction

Incontinentia Pigmenti (IP; OMIM#308300) is a rare multisystemic genomic disorder (1.2/100000 birth prevalence in European data) (1). This X-linked dominant disorder is usually lethal in males and affects the neuroectodermal tissues in females that survive due to a skewed X-chromosome inactivation (XCI) profile (2). IP is caused by *IKBKG/NEMO* gene mutations, encoding a regulatory component of the inhibitor of kappa-B kinase (IKK) complex, which lead to loss of nuclear factor kappa-light-chain-enhancer of activated B cell (NF-κB) activation and increase cell death (3). The recurrent pathogenic variant in individuals with IP is an 11.7 kb deletion of exons 4 through 10 of *IKBKG/NEMO* [NC_000023.11. (154556377_154558531)(154565046?) del; NG_009896.1.399-?_1260+?del] named *NEMOdelta4_10*. It is caused by non-allelic homologous recombination (NAHR) between MER67B repetitive sequences (2). IP clinical diagnosis, generally at birth, is based on the characteristic progression of cutaneous lesions: *vesiculopustular* (Stage 1), *papular verrucous* (Stage 2), *hyperpigmented* (Stage 3), and *hypopigmented* (Stage 4) lesions (2). In addition to dermatologic findings, ocular (retinal hypervascularization, strabismus, cataracts, optic atrophy, retinal pigmentary abnormalities, and microphthalmia) (4), neurological (seizures and intellectual disability) (5–10), myelination delays and ventricular dilatation (5), and other CNS abnormalities have been reported in approximately 27 and 30% of individuals with IP, respectively (5, 10). Teeth defects are also reported in >40% of cases (7, 10, 11). Recent studies have identified neutralizing autoantibodies against type I interferons (AAN-I-IFNs) in women with IP, which may increase the risk of severe viral infections, including COVID-19 and influenza (12–14).

Mediator complex subunit13-like (MED13L)-related intellectual disability (ID) (MRFACD; OMIM#616789; 70 cases reported) (1) is caused by *MED13L* gene mutations inherited in an autosomal-dominant pattern (15, 16), and patients have a broad range of clinical manifestations (17). As reported, 100% of patients had ID, speech delay, and motor delay (17); 72% had hypotonia and anomalies of hands and feet; and slightly less than half of the patients had cerebral Magnetic Resonance Imaging (MRI) abnormalities (18). Ophthalmological abnormalities were described in 39% of patients, autistic features in 28% of patients, and complex congenital heart defects in 25% of patients (19). Moreover, facial dysmorphisms (bulbous nasal tip, depressed/broad nasal bridge, open-mouth appearance, and low-set ears) were observed in more than half of the patients (17, 19).

In this study, we describe the case of a female infant with a clinical presentation of IP at birth who carried postzygotic mosaicism for the genomic deletion of the *IKBKG* gene (*NEMOdelta4_10*) and a *de novo* deleterious variant in the *MED13L* gene (*MED13L*: c.1708_1709del). We propose that the presence of these two rare pathogenic variants underlies the complex clinical presentation, underscoring the utility of comprehensive genomic investigation in diagnostically challenging cases.

## Methods

### Family trios, samples, and ethics statement

The proband and her parents were included in the Incontinentia Pigmenti Genetic Biobank (IPGB)^1^ after obtaining signed, informed consent from the participating individuals. Clinical information was collected by using the clinical IP questionnaire developed by the Incontinentia Pigmenti International Foundation (IPIF)^2^ and the Italian Association of Incontinentia Pigmenti (IPASSI)^3^ and is available upon request. Samples (DNA and serum) were collected and stored in the IPGB biobank (see Footnote 1). The IPGB is accredited by the Italian network BBMRI-ERIC (European Research Infrastructure for Biobanking and Biomolecular Resource). This study was conducted in accordance with the IPGB project, approved by the Ethics Committee of Federico II University (protocol code: 45/15ES1 and date of approval: 21 December 2020).

ChatGPT was used solely for language editing.

### Genetic analysis

The detection of the genomic deletion, *NEMOdelta4_10,* at the IP *locus* was performed (20). Quantification of the ratio between the *NEMOdelta4_10* allele and the *IKBKG* wild-type allele in the patient and in the control was performed by using ImageJ^4^ on four independent analyses.

Droplet digital PCR (ddPCR) reactions were prepared according to the manual instruction: 0.5 μl of template (10 ng/μl genomic DNA), 11 μl of 2X ddPCR EvaGreen Supermix 2X #1864033 (Bio-Rad) (no dUTP), and 1 μl of oligonucleotides (final concentration of 150 nM of each primer) in a total volume of 22 μl. Droplets were generated using the QX200 droplet generator. After droplet generation, 40 μl of droplets containing the reaction was transferred to the 96-well plates and resealed using the plate sealer. PCR amplification was performed using endpoint PCR conditions: 95 °C for 5 min (hot start polymerase activation), followed by 40 cycles at 95 °C for 30 s, annealing at 58 °C for 1 min, and a final deactivation step at 90 °C for 5 min. All the steps were performed with a ramp rate of 2 °C/s.

The ddPCR amplification outcome was analyzed using the QX600 Droplet Digital PCR System and QX Manager software 2.0 (Bio-Rad).

Quantitative PCR analysis (qPCR) was performed on the Applied Biosystems 7900HT Real-time PCR using the SYBR green system and primers P5, P6, P7, P8, and P9 (20). For each sample and control, the relative amount of each amplicon was determined as Mean Normalized Expression (MNE) *versus* a healthy male control. The expression was normalized with respect to the internal control *β2 microglobulin* (*B2M* gene, GenBank NM_004048.2; OMIM #109700). A standard curve was generated by serial dilutions of DNA obtained from peripheral blood of an IP female patient carrying the constitutive *NEMOdelta4_10* allele with DNA from a healthy female control to obtain samples with different percentages of the deleted allele (25, 16.7, 12.5, 8.3, and 0%).

X-chromosome inactivation was assessed by a method that analyses methylation of the Human Androgen Receptor Assay (HUMARA) locus (21). The skewing ratio was calculated by applying the algorithm. Ratios less than 80:20 were considered a random XCI, ratios 80:20–90:10 were considered a moderate skewing index, and ratios >90:10 were considered a skewing index.

### Clinical exome sequencing (clinical-ES)

Library preparation of samples was performed following the SureSelectQXT Automated Target Enrichment protocol for the Illumina platform (Version B0, November 2015, Agilent Technologies). The trio was enriched using the SureSelect Custom Constitutional Panel (17 Mb), and enriched DNA was validated and quantified by microfluidic analysis using the High Sensitivity D1000 ScreenTape Assay and the 4,200 TapeStation System (Agilent Technologies). Libraries were sequenced on the NovaSeq 6,000 system with paired-end runs of at least 2×150 nt (Illumina Inc.). Sequence data were analyzed using an in-house automated pipeline combining commercial and custom tools. The average exome coverage was at least 100X, with 90% of bases covered by at least 40 reads. Variant detection assumed an autosomal dominant inheritance model, focusing on variants with a minor allele frequency ≤0.001 in gnomAD, dbSNP, ExAC, ClinVar, and an internal database of ~5,400 Italian subjects.

### Autoantibody detection

Screening of neutralizing activity toward type I interferon was performed as previously described at the Laboratory of Human Genetics of Infectious Diseases, Necker Hospital for Sick Children, Paris, France, and University of Paris, Imagine Institute, Paris, France (12–14). Briefly, neutralizing anti-IFN-I (α2 and *ω*) autoantibodies were assessed using a luciferase reporter assay. HEK293T cells were co-transfected with an ISRE-firefly luciferase plasmid (pGL4.45) and a Renilla luciferase plasmid (pRL-SV40) using Lipofectamine™ 3,000. After 24 h, the cells were incubated in Dulbecco’s Modified Eagle Medium (DMEM) with 2% fetal bovine serum (FBS) and 10% heat-inactivated serum/plasma (1:10 dilution) and either left unstimulated or treated with IFN-α2 or IFN-ω (10 ng/mL or 100 pg/ml) for 16 h. Luciferase activity was measured using the Dual-Luciferase Reporter Assay System, and normalized firefly/Renilla values were expressed as a percentage of the median response in healthy controls. Samples were neutralized if dual-luciferase activity was below 3 (13).

## Results

### Case report: clinical features

The female patient (II:1, Figure 1A) was born at term after 40 + 5 weeks of pregnancy with a birth weight of 3,000 g, a length of 48.5 cm (3rd centile), and a head circumference of 33.5 cm (third—tenth centile). She did not have a history of asphyxia at birth (Apgar scores were 8/10) and any complications throughout the pregnancy. She was the first child of healthy non-consanguineous parents, and her mother had three first-trimester miscarriages (I:2, Figure 1A). The patient was conceived naturally; there was no history of assisted reproductive technology.

**Figure 1:**
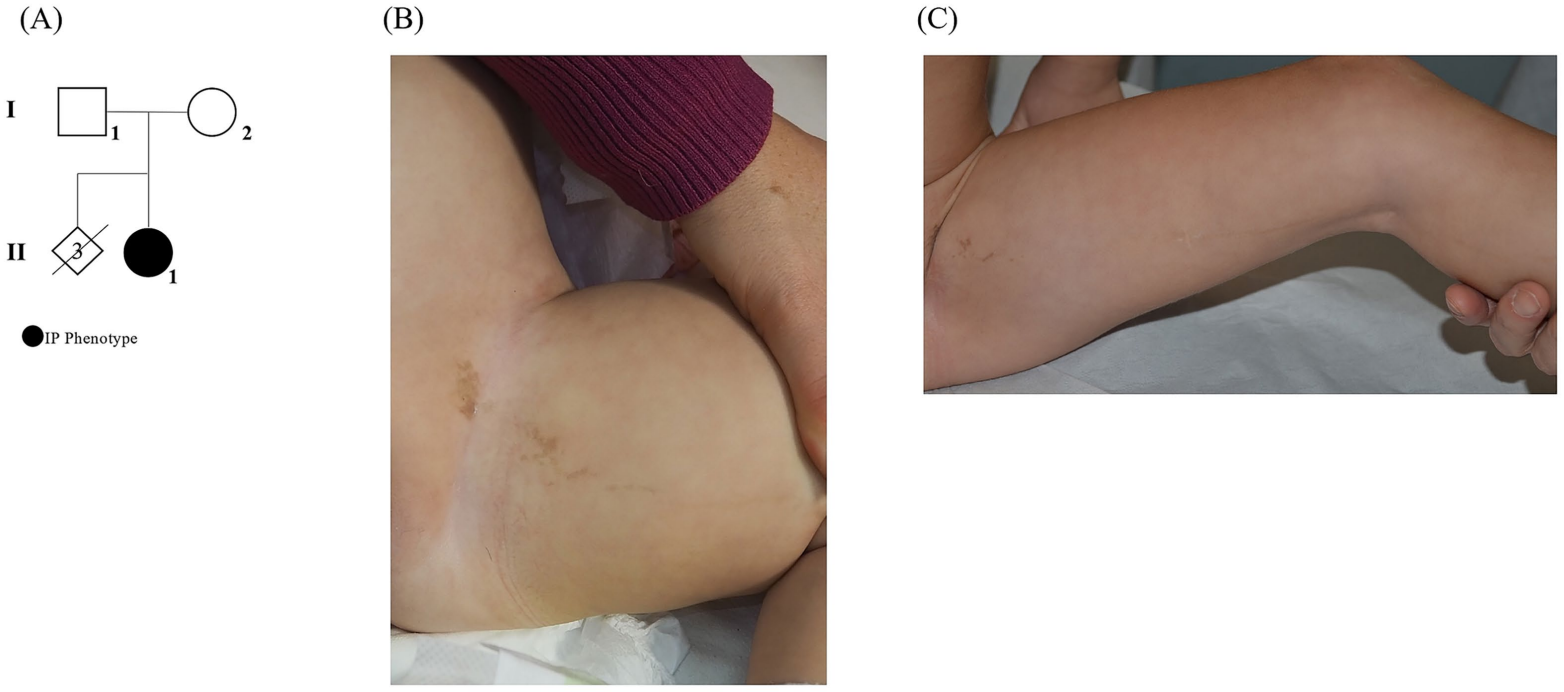
Family pedigree and IP skin features of the patient. Pedigree of family trios: I:1_father; I:2_mother; II:1_proband; the number “3” in the rhombus symbol indicates three miscarriages in the first trimester of pregnancy **(A)**. Linear pigmentation on the left groin and lower limb at 7 months **(B)** and at 3 years old **(C)**.

Forty-eight hours after birth, the infant exhibited vesiculopustular skin lesions restricted to the left lower limb that evolved into hyperkeratotic lesions by 3 months of age, followed by pigmented macules at 5 months and hyperpigmented linear macules at 7 months, always restricted to the left lower limb (Figures 1B, C). At 4 months, the patient presented with moderate strabismus of the right eye and an impaired adduction of the left eye, diagnosed as Duane syndrome due to a 6th cranial nerve development defect. At 6 months, neurological assessments indicated hypotonia and early signs suggesting moderate intellectual disability. Magnetic resonance imaging (MRI) at 7 months revealed lateral ventricle enlargement. At 21 months, she showed motor, developmental, and speech delays with moderate intellectual disability (ID) and poor communication skills. Finally, there was no history of recurrent viral infection (Table 1), and neither alopecia nor dental abnormalities were detected. Moreover, a skin biopsy was performed and showed orthokeratosis with mild acanthosis and papillomatosis of the epidermis and apoptotic keratinocytes. She was diagnosed with an IP phenotype.

**Table 1:** Timeline of clinical presentation of the patient during the period of 0–21 months.

Time	Clinical presentation
48 h at birth	Blistering and vesiculopustular lesions only at the left lower limb.
3 months	Wart-like rash and hyperkeratotic lesions only at the left lower limb.
4–5 months	Linear hyperpigmentation only at the left lower limb. Moderate strabismus of the right eye. Deficiency of left eye adduction with VI cranial nerve deficit.
6–7 months	Linear hyperpigmented macules only at the left lower limb. Hypotonia. Moderate intellectual disability. MRI: incomplete myelination and supratentorial ventricular dilatation with a squared configuration
21 months	Motor delay. Developmental delay. Speech delay. Moderate intellectual disabilities. Poor communication skills. No history of recurrent viral infection.

MRI, magnetic resonance imaging.

### Genetic testing in DNA from different tissues revealed mosaicism for the mutated allele in the IKBKG gene and a constitutive mutation in the MED13L gene

**Figure 2:**
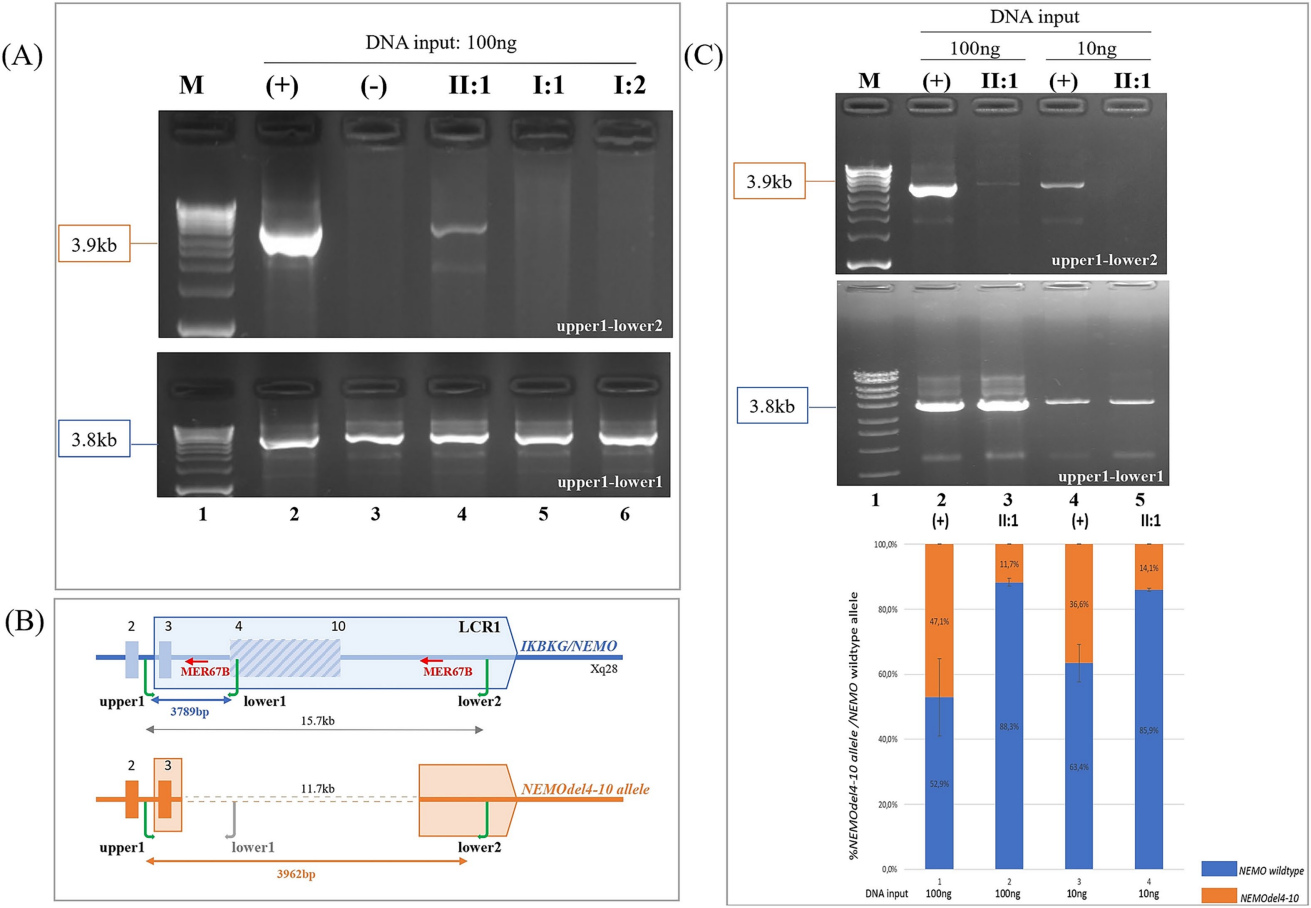
Targeted PCR analysis by long-range PCR products corresponding to amplification of the *NEMOdelta4_10* allele. Representative structure of the *IKBKG/NEMO* gene in a female cell heterozygous for the *NEMOdelta4_10.* Blue represents the *IKBKG/NEMO* wild-type allele, and orange represents the *NEMOdelta4_10* allele. PCR amplification with primers upper1 (before LCR1) and lower1 (between exons 4 and 10) consistently yields a 3.8-kb product, identifying the wild-type allele. In contrast, when using upper 1 and lower 2 (downstream of exon 10 and MER67B repeat), the wild-type allele does not amplify due to the large fragment size (~15.7 kb). However, this setup successfully amplifies a 3.9 kb product from the *NEMOdel4_10* allele, which is missing the 11.7 kb region between the two MER67B elements **(A)**. Long-range PCR tests, specific for the *NEMOdelta4_10* allele (upper 1 and lower 2) and for the wild-type allele (upper 1 and lower 1), respectively, were performed on DNA from the IP control female (+) (lane 2), healthy male control (−) (lane 3), patient II:1 (lane 4), and the patient’s parents (I:1, I:2) (lanes 5 and 6) **(B)**. Long-range PCR tests, specific for the *NEMOdelta4_10* allele and for the *IKBKG*/*NEMO* wild-type allele on different DNA inputs (100 ng, lanes 2–3, and 10 ng, lanes 4–5) of IP female positive control (+) (lanes 2 and 4) and proband II:1 (lanes 3 and 5). Densitometric analysis by using ImageJ (https://imagej.nih.gov/ij/download.html) expressing the % *NEMOdel* allele (orange) and the wild-type allele (blue) in the patient (II:1; lanes 3 and 5) and in the control (+; lanes 2 and 4) in the upper gel **(C)**. M, marker DNA size.

Targeted PCR analysis of the genomic DNA of the patient’s peripheral blood mononuclear cells (PBMCs) showed the presence of genomic deletion *NEMOdelta4_10* [NC_000023.11:g.(154556377_154558531)(154565046_?)del] in the IP *locus* (Figure 2A). *NEMOdelta4_10* represents the deleted allele of the region from exon 4 to exon 10 of the *IKBKG* gene and causes IP in 80% of affected patients (2). In addition, densitometric analysis estimated that the diagnostic PCR product corresponding to amplification of the *NEMOdelta4_10* allele (3.9 kb, Figure 2B) in the patient (II:1) was on average 22% (0.22 ± 0.016) *vs* 50% (0.50 ± 0.047) in the IP female control (+) (Figure 2C). This result was suggestive of cellular blood mosaicism due to the presence of cells that were homozygous for alleles without deletion (wild type) and cells heterozygous for the *NEMOdelta4_10* allele.

Droplet digital PCR (ddPCR) and quantitative real-time PCR (qPCR) confirmed mosaicism for the *NEMOdel4–10* (Supplementary Figures 1 and 2C, respectively).

Using allele-specific probes, P6–P7 previously described (20), we tested the mosaic ratio of the *NEMOdelta4_10* allele (variant allele frequency, VAF) in DNA from blood, buccal swab, and urine samples, and we detected a mosaic ratio of the *NEMOdelta4_10* allele of 10.4% in blood and 12.5% in urine *vs* 25% in blood control IP female patients, and it was absent in buccal swab (Supplementary Figures 2A,B). This corresponded to cellular mosaicism of ~40% in blood and ~50% in urine for the deletion (Supplementary Table 1), consistent with postzygotic somatic mosaicism. Of note, neither the patient nor the parents carried risk alleles for the generation of *de novo NEMOdelta4_10* (*NEMOPdel4-10* or *MER67Bdup* alleles) (22).

Consistent with the hypothesis of mosaicism in female IP patients (23, 24) (Supplementary Table 2), the XCI, performed by the HUMARA on DNA from peripheral blood, showed a random XCI (60:40) profile in the patient.

Since analysis of affected and unaffected skin biopsies would have been invasive for the patient and, more importantly, would not have influenced clinical decision-making, we did not investigate the skin. Instead, we considered whether the severity of the proband’s phenotype could be attributed exclusively to the mosaic *NEMOdelta4_10* mutation or whether additional genomic variants in the patient’s background might have contributed to the exacerbation of the clinical manifestations. Furthermore, genetic analysis was performed by trio Clinical Exome Sequencing (Clinical-ES) in the known genes (5,228 tested genes) associated with diseases. We found a pathogenic *de novo* variant in the *MED13L* gene (NM_015335.5: c.1708_1709del; p.Ser570fs*27) (Supplementary Figure 3), resulting in a premature stop codon and a truncated protein. The VAF was consistent with a constitutive heterozygous germline variant. We observed 44.4% of sequencing reads carrying the mutation (59 mutated reads out of 133 total), which fell within the expected range for heterozygosity and did not support a mosaic pattern for this variant. Moreover, 100% of sequencing reads of both parents (the mother reads a total of 43 wild types; the father reads a total of 39 wild types) confirmed a *de novo* status of the mutation in the patient.

This mutation is located on chromosome 12 at positions 116,008,704–116,008,705 (GRCh38), was previously reported as pathogenic in intellectual disability (ClinVar: RCV001257601.3, VCV000221555.8), is absent in gnomAD, and has been reported in three previously described cases (25–27).

### No detectable autoantibodies against type I IFNs

To investigate the presence of autoantibodies against type I IFNs, the proband’s serum (collected at age 21 months) was tested for neutralizing activity against IFN-α2 and IFN-*ω* at either high (10 ng/ml) or low (100 pg/ml) cytokine concentrations using a Dual-Luciferase Reporter [DLR] assay (13). No neutralizing activity was detected: at high concentrations, the DLR values were 219.40 and 149.41 for IFN-α2 and IFN-ω, respectively; at low concentrations, the DLR values were 44.47 and 17.61 for IFN-α2 and IFN-ω, respectively, thus non-neutralizing. Overall, the patient was negative for the presence of neutralizing autoantibodies to type I IFNs.

## Discussion

The occurrence of two genetically unlinked rare disorders in a patient born to non-consanguineous parents is an extremely unusual condition, especially when two *de novo* mutational events occur in the same individual: one producing postzygotic mosaicism in the *IKBKG* gene and the other causing a constitutive mutation in the *MED13L* gene.

The patient presented at birth with the clinical signs of IP disease, and, although the molecular diagnosis confirmed the IP disease (presence of *NEMOdelta4_10* mosaicism in the *IKBKG* gene), during development, the neurological clinical signs appeared only partially consistent with an exclusive IP diagnosis, suggesting the need for further genetic investigation. The hypothesis of multiple genetic causes was supported by the identification of a second *de novo* pathogenic variant in another locus, *MED13L* (NM_015335.5:c.1708_1709del). These findings indicated a complex phenotypic presentation characterized by clinical signs of IP disease, such as skin lesions exclusively attributed to *NEMOdelta4_10* mosaicism (2); *MED13L*-related intellectual disability exclusively attributed to the *MED13L* mutation, NM_015335.5:c.1708_1709del (p. Ser570fs*27), such as speech delay (17, 19) and hypotonia (26, 27) (Supplementary Table 3); and both diseases, as the neurological and ophthalmological findings, such as developmental delay, intellectual disability, enlarged bilateral lateral ventricles, and strabismus (Table 1), could reflect the contribution of both genetic alterations.

Indeed, skin lesions were reported in all IP patients (2) but never in *MED13L*-related patients. Conversely, severe speech delay was reported in 98% of MED13L-related patients (17, 19) but never in IP patients. However, ID (2, 5–9, 17, 19), motor delay (5, 19), and ocular anomalies (7, 17) are reported at different percentages in both diseases (Supplementary Table 4). Based on these considerations, establishing a clear genotype–phenotype correlation is challenging in this reported case.

*MED13L* haploinsufficiency is a well-recognized cause of neurodevelopmental impairment, including intellectual disability, hypotonia, and delayed motor and speech development (15). Initially described as a complex disorder, it has been recently redefined as a syndromic ID that encompasses a distinctive set of features, including dysmorphic facial features, ID, and speech impairment (impaired intellectual development and distinctive facial features with or without cardiac defects; OMIM# 616789), with variable penetrance for congenital heart defects, inherited in an autosomal dominant manner (15). MED13L-related intellectual disability is caused by *MED13L* gene mutations that map to chromosome 12q24.21. The gene encodes a subunit protein of the large mediator complex, which is involved in gene transcription mediated by transcription factors and RNA polymerase II (28). The vital role of *MED13L* in the regulation of the neural crest cells and neurogenesis, along with its morphological and functional impacts on cortical neurons, may exert great influence on intellectual development (29–31).

Central nervous system involvement in Incontinentia Pigmenti is known to be variable and observed in approximately 30% of patients with constitutive *IKBKG* mutation (2, 5–9). Given the role of *IKBKG* in NF-κB signaling, which is implicated in neuronal development and survival, a contributory effect of *NEMOdelta4_10* mosaicism on the neurological and ophthalmological phenotype may be taken into consideration. Indeed, it has been reported in 5/12 (41.6%) (32, 33) IP male patients with *NEMOdelta4_10* mosaicism (Supplementary Table 2) (20, 32–34). Moreover, in the context of genetic mosaicism, the resulting phenotype is difficult to predict, as the biological consequences of a mutation are mainly determined by its developmental timing, as well as the type and distribution of the affected tissue. Consequently, the neurological features observed in this patient are so largely consistent with the MED13L-associated phenotype that it is likely possible that *IKBKG* mosaicism modulates the severity or variability of the manifestations in this patient. The insufficient mechanistic integration between the two mutated genes does not provide support for the additive effect in the patient described in this study, so the effects of concurrence of these mutations at a functional level remain unknown and only speculative.

Notably, the absence of anti-IFN-I autoantibodies in this patient does not rule out the possibility that they may develop later in life, emphasizing the need for longitudinal follow-up by testing for autoantibodies against type I IFNs every year or 2 years. If positive, extensive vaccination (except live vaccines) against flu, Varicella-Zoster Virus (VZV), COVID-19, and Respiratory Syncytial Virus (RSV) would be recommended.

This genetic asset, never previously reported, although it is not supported by functional findings, further underscores the challenge of predicting complex phenotypes and strongly suggests the need to improve the care of patients with rare and complex phenotypes by integrating classical molecular diagnostics with the investigation of additional genetic variants to provide appropriate counseling and clinical management.

## Data Availability

The datasets presented in this article are not readily available because data is available but not submitted in a dataset, as raw data can be shared. Requests to access the datasets should be directed to Francesca Fusco, francesca.fusco@igb.cnr.it.

## Footnotes

1
http://www.igb.cnr.it/ipgb/

2
http://www.ipif.org/ip_consortium.html

3
http://www.incontinentiapigmenti.it/

4
https://imagej.nih.gov/ij/download.html
