# Supplementary material for: A case of Incontinentia Pigmenti associated with concurrent IKBKG/NEMO and MED13L mutations
Source: Front Med (Lausanne). 2026 Jun 18;13:1819035. doi: 10.3389/fmed.2026.1819035 (PMC13322796; doi:10.3389/fmed.2026.1819035)
Supplement: Supplementary file 1 [file Data_Sheet_1.docx]

***Supplementary Material***

**Supplementary Figures:**

**Supplementary Figure 1**


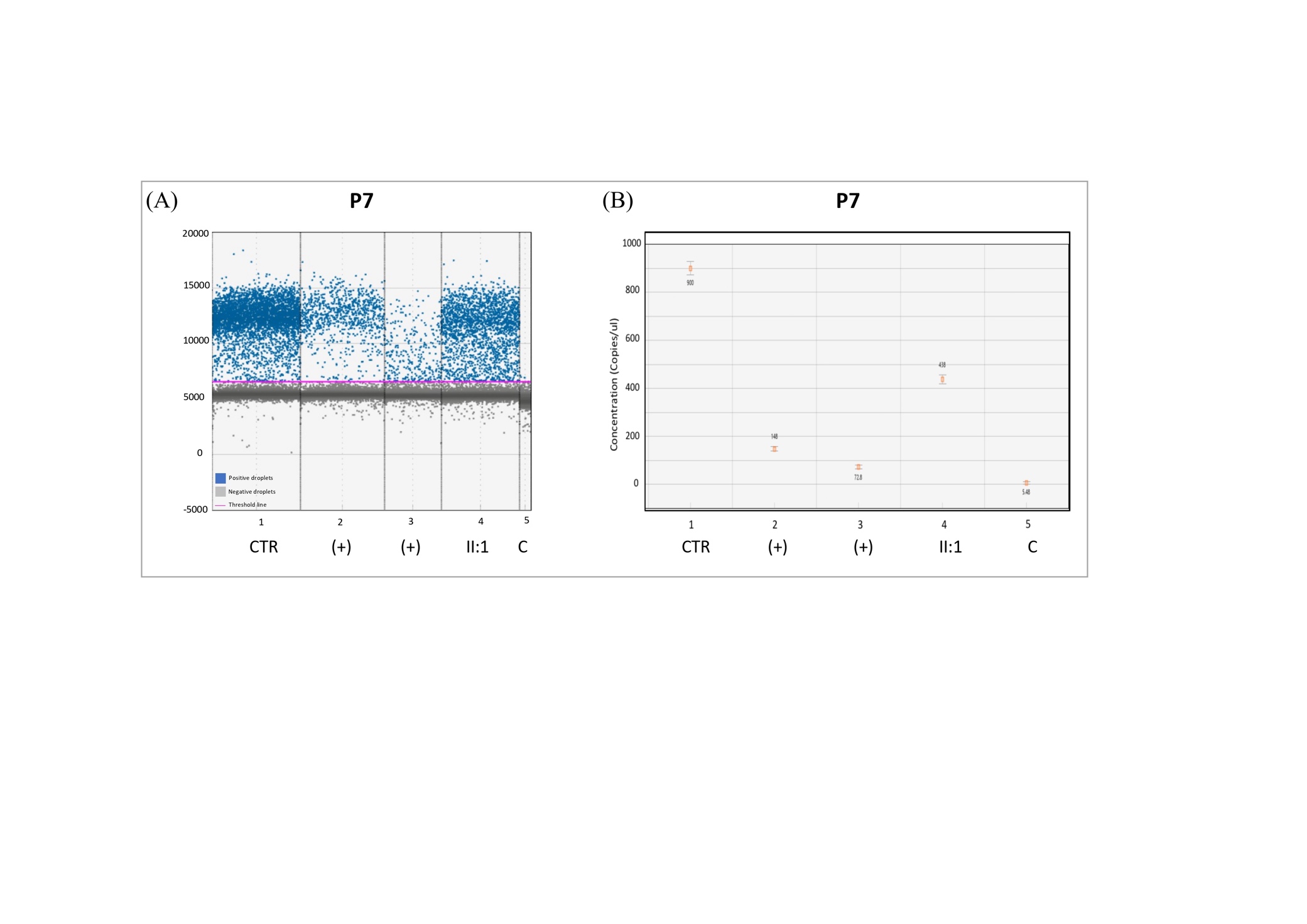


**Supplementary Figure 1:** Absolute quantification of the DNA target (P7, primers located between exon4-10, identifying the copy number variation in the presence of *NEMOdelta4_10)^1^* across different samples : Lane 1 (CTR), representing a healthy female exhibiting a concentration of 900 copies/µL; lane 2-3 (+) representing two different IP females carrying the *NEMOdelta4_10* allele showed on average 110.4+53.17 copies/µL; lane 4 (II:1) representing our patient (II:1) sample showing 438 copies/µL. Lane 5(C), ddPCR negative control.

The absolute concentration of the target (P7) was calculated based on the fraction of positive droplets, using Poisson correction as implemented in the QXmanager software 2.0 (Bio-Rad). The value P7 observed in (II:1) suggests a mosaicism condition in the patient.

Negative controls showed no detectable amplification, confirming assay specificity. The clear separation between positive and negative droplet populations was evident in the fluorescence amplitude plots.

**Supplementary Figure 2**


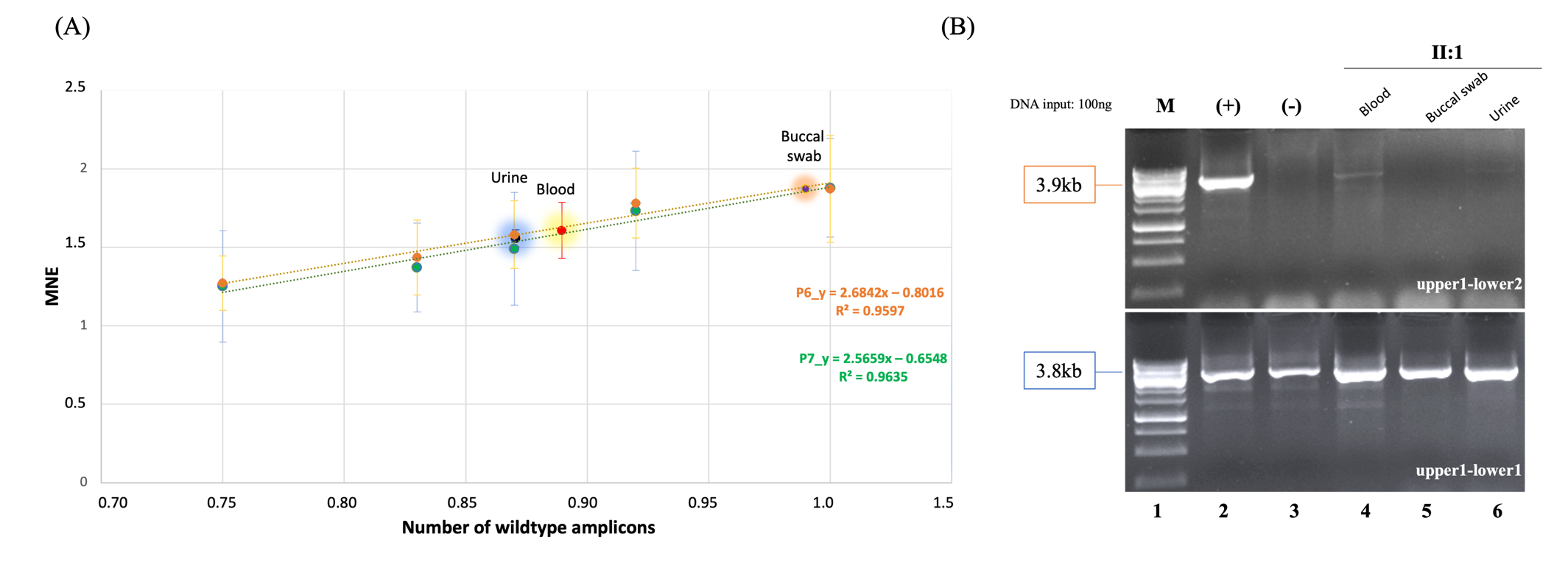


**Supplementary Figure 2:** Quantitative real-time PCR (qPCR) analysis for indirect determination of *NEMOdelta4_10* in IP patient (II:1).

Standard curves with P6 (green) and P7 (orange) primers, and quantification of *IKBKG* wildtype allele by crossing points with regression line for P6 [y = 2.6842x -0.8016 R² = 0.9597] and for P7 [y = 2.5659x -0.6548 R² = 0.9635].

The data were normalized with the internal control *B2M* gene and expressed as expressed as MNE (2^–∆∆Ct^). Number of wildtype amplicons are reported in Supplementary Table 1. The primers P6 and P7 identify the copy number variation in the presence of *NEMOdelta4_10^1^*.

Yellow circle= blood sample; blue circle= urine samples and orange circle= buccal swab samples (A).

Long-range PCR assays specific for the *NEMOdelta4_10* allele (upper1/lower2) and for the wild-type allele (upper1/lower1) were performed on DNA from a female IP-positive control (+) (lane 2), a healthy male control (lane 3), and tissues from patient II:1 (lanes 4–6): blood, buccal swab and urine, respectively (B).

**Supplementary Figure 3**

**Supplementary Figure 3**: The point mutation of *de novo* variant in *MED13L* gene validated by Sanger sequencing. The *MED13L c.1708_1709del* mutation in Patient (II:1). The father (I:1) and the mother (I:2) are wildtype.

**Supplementary Tables:**

**Table 1. Quantification values of the amplicons located in the IP locus in duplicated region LCRs (P5, P6, P7, P8) and outside the LCRs (P9) in the sample from blood of IP patient (I:1)**

**(A)**

| DNA samples | Genotype | P5 | | P8 | | P9 | |
| --- | --- | --- | --- | --- | --- | --- | --- |
|  |  | MNE | N | MNE | N | MNE | N |
| Healthy Female | X_X | 2.070+0.07 | 4 | 2.68+0.49 | 4 | 2.36+0.30 | 2 |
| IP Female | X_X*_NEMOdel_* | 1.710+0.001 | 4 | 1.25+0.09 | 4 | 1.82+0.03 | 2 |
| IP Patient (II:1) blood |  | 1.820 ± 0.07 | 4 | 1.69+0.50 | 4 | 1.99 ± 0.07 | 2 |
| IP Patient (II:1) buccal swab |  | 2.465+0.07 | 4 | 2.093+0.04 | 4 | 1.369+0.08 | 2 |
| IP Patient (II:1) urine |  | 1.437+0.13 | 4 | 1.789+0.06 | 4 | 1.557+0.10 | 2 |

N=number of amplicon alleles/cell. The interval [minimum value – maximum value] threshold for each probe (P) is reported as Threshold and alleles: P5(4)= [0.91-1.81], P5(2)=[0.30-0.50], P8(4)=[0.90-1.62], P8 (2)=[0.42-0.59], P9 (2)=[0.81-1.90], P9(1)=[0.40-0.77]. The data were normalized with the internal control *B2M* gene and expressed as 2^-∆∆Ct^ MNE=normalized data respect to male control.

**(B)Standard Curve**

| DNA samples | GenotypE (*) | P6 MNE | P7 MNE | N | % *NEMOdelta4_10* |
| --- | --- | --- | --- | --- | --- |
| IP Female *NEMOdelta4_10* | X_X*_NEMOdel_*  (1:0) | 1.251+0.35 | 1.271+0.17 | 0.75 (3:4) | 25.0% |
| IP Female *NEMOdelta4_10* /Female Control | X_X*_NEMOde_*_l_ /XX (2:1) | 1.371+0.28 | 1.435+0.24 | 0.83 (10:12) | 16.7% |
| IP Female *NEMOdelta4_10* /Female Control | X_X*_NEMOdel_* /XX (1:1) | 1.490+0.36 | 1.580+0.22 | 0.87 (7:8) | 12.5% |
| IP Female *NEMOdelta4_10* /Female Control | X_X*_NEMOdel_* /XX (1:2) | 1.732+0.38 | 1.781+0.22 | 0.92 (11:12) | 8.3% |
| Female Control | XX (0:1) | 1.878+0.31 | 1.871+0.34 | 1.00 (4:4) | 0% |
| IP Patient (II:1) blood |  | 1.429+0.02 | 1.785+0.001 | 0.89**+0.20 (~11:12) | ~12.5-8.3% (10.4%) |
| IP Patient (II:1) buccal swab |  | 1.821+0.50 | 1.919+0.39 | 0.99**+0.02 (~4:4) | 0% |
| IP Patient (II:1) urine |  | 1.502+0.55 | 1.612+0.16 | 0.87**+0.24 (~7:8) | ~12.5% |

* Serial dilutions obtained by mixing DNA from IP Female *NEMOdelta4_10* heterozygous and DNA from healthy Female control (no deletion allele). N=number of Observed: Expected amplicons. **Average between Crossing points of patient (II:1) obtained by interpolating on standard curve equations of P6 [y=2.5659x – 0.6548 R² = 0.9635] and P7 [y=2.6842x – 0.8016 R² = 0.9597], respectively. The primers P6 and P7 identify the copy number variation in the presence of *NEMOdelta4_10*^1^. IP Female *NEMOdelta4_10=*IP female carrying the constitutive *NEMOdelta4_10* allele. Female Control=healthy Female Control. MNE = the value expressed in 2^–∆∆Ct^ normalized with the internal control *B2M* gene, and respect to male control. X*_NEMOdel=_*X-chromosome carrying *NEMOdelta4_10* allele.

**Table 2. Clinical features of IP patients carrying the mosaicism of *NEMOdelta4_10* mutation and our patient (II:1)**

|  | Fusco et al., 2007^2^ | Fusco et al., 2017^1^ | Alabdullatif et al., 2018^3^ | Haque et al., 2020^4^ | Kawai et al., 2020^5^ | Shibata et al., 2021^6^ | *this paper* |
| --- | --- | --- | --- | --- | --- | --- | --- |
| Gender | Three males | male | Six males | male | Four females and one male | female | female |
| Time of diagnosis | (1)1y; (2)9m; (3)8y | 34y | (1)9m; (2)11m; (3)2m; (4)15d; (5)2.5m; (6)3m | 4m | Females: (1)2y, (2)0y, (3)0y, (4)0y and (5)0y male | 2days at birth | at birth |
| %Mosaicism detected | Blood, Skin biopsy | Urine (25%)  Germline (35%) | Blood in five, skin biopsy in one. | Saliva (45%) | Blood low level of mosaicism  XCI 61.9% | blood low level of mosaicism  XCI no skewed | blood ~40%  urine ~50%  XCI 60% |
| Skin lesions | (1)1m; 2m; 4m; 1y (R leg-foot).  (2)1m;3m; (L arm, legs, trunk).  (3)1m; 10m; 6y (limbs, hands, abdomen). | Infantile age; 12y (trunk, limbs, back) | (1)Head, trunk, arms, legs  (2)Trunk, legs, right arm  (3)Legs  (4)Arms, legs  (5)Head, legs  (6)Head, trunk, arms, legs | blistering rash, vesicles and pustules | skin alterations | blisters on right forearm, abdomen, and thigh. | skin lesions on left lower limb |
| Ophthalmic anomalies | (1) Ocular defects.  (2) Not reported.  (3) Not reported. | Myopia | (1) Papillary, hypoplasia, retinal detachment, amblyopia (right eye)  (2) Hypermetropia  (3) Retinal vasculopathy  (4)(5)(6)Not reported | Retinopathy at 2 weeks of age | Not reported | No ophthalmic defects | moderate strabismus of right eye and an impaired adduction of left eye. |
| Neurologic anomalies | (1) CNS defects.  (2) Not reported.  (3) Not reported. | Not reported | (1)Global hypotonia, CWML  (2)Right hemiplegia, epilepsy, learning difficulties  (3)CWML  (4)CWML, epilepsy, learning difficulties  (5)(6)Not reported |  |  | No neurologic defects. | Hypotonia; moderate ID; MRI: lateral ventricles enlargement. Motor, developmental |
| other defects | (1) Nail defects.  (2) Not reported.  (3) Dental defects. | Nail defects;  One cone-shaped tooth | Not reported | malformed teeth |  |  | Speech delays |

d=days; m= months; y=years; CWML =Cerebral white matter lesions seen on magnetic resonance imaging (MRI); Nd_not detected; L_Left; R_Right; ID_intellectual disability

**Table 3. Clinical features of patients carrying the *MED13L* *c.1708_1709del* (p.Ser570fs*27) mutation and our patient (II:1)**

|  | Hamdan et al., 2014^7^ | Codina-Solà et al., 2015^8^ | Ewans et al., 2018^9^ | *this paper* |
| --- | --- | --- | --- | --- |
| Time of diagnosis | 5 years-old | 16 years-old | 7years-old | 21-months |
| Inheritance | AD inheritance; *de novo* | AD inheritance; *de novo* | AD inheritance; *de novo* | AD inheritance; *de novo* |
| Intellectual Disability | moderate global | severe | reported | moderate global |
| Hypotonia | not reported | reported | reported | reported |
| Impaired language development | At the age of 5 years, she could make short, incomplete sentences | reported | not reported | reported |
| Brain Resonance Imaging (MRI) findings | Brain CT scan performed at the age of 2 years showed mildly increased extra-axial CSF spaces | not reported | not reported | MRI showed enlarged bilateral lateral ventricles |
| Strabismus | reported | not reported | not reported | reported |
| Other features | overweight | umbilical hernia | cleft palate | Duane syndrome |
| Dysmorphic features | not reported | reported | not reported | not reported |
| Autistic features | excluded | reported | not reported | not reported |
| Sleep disturbance | not reported | reported | not reported | not reported |
| Cardiovascular anomalies | excluded | not reported | not reported | excluded |

AD= Autosomal Dominant inheritance

**Table 4: Clinical features of IP and MRFACD**

|  | IP  MIM#308300 | MRFACD  MIM#616789 |
| --- | --- | --- |
| Skin lesions | 100%^10^ | 0% |
| Intellectual Disability | 26%^10-14^ | 100% ^15,16^ |
| Severe speech delay | 0% | 98% ^15,16^ |
| Hypotonia | 0%*^17,18^ | 63-72% ^15,16^ |
| Motor delay | 25.7%^12^ | 99%^16^ |
| Strabismus | 8.9%^19^ | 31% ^15^ |
| Optical nerve atrophy | 1.7% ^19^ | 0% |
| Teeth defects | 48.9%^20,21^ | 0% |
| anti-IFN-I autoantibodies | 36%^22^ | Not reported |

*reported two cases in Peebles et al., 2021;^17^ Miteva et al., 2001^18^

**SI References**

1. Fusco F, Conte MI, Diociaiuti A, Bigoni S, Branda MF, Ferlini A, et al. Unusual Father-to-Daughter Transmission of Incontinentia Pigmenti Due to Mosaicism in IP Males. Pediatrics (2017) 140: e20162950
2. Fusco F, Fimiani G, Tadini G, Michele D, Ursini MV. Clinical diagnosis of incontinentia pigmenti in a cohort of male patients. J Am Acad Dermatol. (2007) 56:264-267. doi: 10.1016/j.jaad.2006.09.019. Epub 2006 Nov 7. PMID: 17224368.
3. Alabdullatif Z, Coulombe J, Steffann J, Bodemer C, Hadj-Rabia S. Postzygotic mosaicism and incontinentia pigmenti in male patients: molecular diagnosis yield. Br J Dermatol. (2018);178:e261-e262. doi: 10.1111/bjd.16092. Epub 2018 Feb 6. PMID: 29077987.
4. Haque MN, Ohtsubo M, Nishina S, Nakao S, Yoshida K, Hosono K, et al*.* Correction to: Analysis of *IKBKG/NEMO* gene in five Japanese cases of incontinentia pigmenti with retinopathy: fine genomic assay of a rare male case with mosaicism. J Hum Genet (2021) 66, 645 <https://doi.org/10.1038/s10038-021-00900-6>
5. Kawai M, Kato T, Tsutsumi M, Shinkai Y, Inagaki H, Kurahashi H. Molecular analysis of low-level mosaicism of the IKBKG mutation using the X Chromosome Inactivation pattern in Incontinentia Pigmenti. Mol Genet Genomic Med. (2020) 8:e1531. doi: 10.1002/mgg3.1531. Epub 2020 Oct 21. PMID: 33085210; PMCID: PMC7767561.
6. Shibata K, Kunisada M, Miyai S, Kawamori S, Kurahashi H, & Nishigori C. Incontinentia pigmenti in a female infant with somatic mosaicism due to the IKBKG variant. The Journal of dermatology (2021) 48: e577–e578
7. Hamdan FF, Srour M, Capo-Chichi JM, Daoud H, Nassif C, Patry L, et al. De novo mutations in moderate or severe intellectual disability. PLoS genetics (2014) 10: e1004772.
8. Codina-Solà M, Rodríguez-Santiago B, Homs A, Santoyo J, Rigau M, Aznar-Laín G, et al. Integrated analysis of whole-exome sequencing and transcriptome profiling in males with autism spectrum disorders. Molecular autism (2015) 6: 21.
9. Ewans LJ, Schofield D, Shrestha R, Zhu Y, Gayevskiy V, Ying K, et al. Whole-exome sequencing reanalysis at 12 months boosts diagnosis and is cost-effective when applied early in Mendelian disorders. Genetics in medicine: official journal of the American College of Medical Genetics (2018) 20: 1564–1574.
10. Scheuerle AE & Ursini MV. Incontinentia Pigmenti. In M. P. Adam (Eds.) et. al., GeneReviews®. University of Washington, Seattle (1999-2025).
11. Meuwissen ME, Mancini GM. Neurological findings in incontinentia pigmenti; a review. Eur J Med Genet. (2012) 55: 323–31.
12. Minić S, Trpinac D, Obradović M. Systematic review of central nervous system anomalies in incontinentia pigmenti. Orphanet J Rare Dis (2013) 8:25–35. doi:10.1186/1750-1172-8-25.
13. Pizzamiglio MR, Piccardi L, Bianchini F, Canzano L, Palerm, L, Fusco F, et al. Incontinentia pigmenti: learning disabilities are a fundamental hallmark of the disease. PloS one (2014) 9: e87771.
14. Pizzamiglio MR, Piccardi L, Bianchini F, Canzano L, Palermo L, Fusco F, et al. Cognitive-behavioural phenotype in a group of girls from 1.2 to 12 years old with the Incontinentia Pigmenti syndrome: Recommendations for clinical management. Appl Neuropsychol Child. (2017) 6:327-334.
15. Campbell AN, Bain J, Doyle SJ. MED13L Syndrome. 2025 Apr 10. In: Adam MP, Bick S, Mirzaa GM, et al., editors. GeneReviews® [Internet]. Seattle (WA): University of Washington, Seattle; 1993-2026. Available from: https://www.ncbi.nlm.nih.gov/books/NBK613517/
16. Cao H,He T, Wang J, Zhou C, Wei X and Zhang X Case Report: Novel mutations in two patients with MED13L-related intellectual disability highlighting the importance of genetic counseling. Front. Genet. (2025) 16:1669849.doi: 10.3389/fgene.2025.1669849
17. Peebles JK, Craddock L, Bennett DD, Longley BJ, Swanson A. Incontinentia Pigmenti: Initial Presentation of Encephalopathy and Seizures. Cutis. (2021) 107:E37-E39. doi: 10.12788/cutis.0227. PMID: 33956621.
18. Miteva L, Nikolova A. Incontinentia pigmenti: a case associated with cardiovascular anomalies. Pediatr Dermatol. (2001) 18:54–56.
19. Minić S, Obradović M, Kovacević I, Trpinac D. Ocular anomalies in incontinentia pigmenti: literature review and meta-analysis. Srp Arh Celok Lek. (2010) 138:408-413.
20. Minic S, Trpinac D, Gabriel H, Gencik M, Obradovic M. Dental and oral anomalies in incontinentia pigmenti: a systematic review. Clin Oral Investig (2013) 17: 1–8.
21. Fusco F, Paciolla M, Conte MI, Pescatore A, Esposito E, Mirabelli P, et al. Incontinentia pigmenti: report on data from 2000 to 2013. Orphanet J Rare Dis. (2014) 9:93.
22. Rosain J, Le Voyer T, Liu X, Gervais A, Polivka L, Cederholm A, et al. Incontinentia pigmenti underlies thymic dysplasia, autoantibodies to type I IFNs, and viral diseases. The Journal of experimental medicine (2024) 221: e20231152.
